# Urban Road Network Expansion and Its Driving Variables: A Case Study of Nanjing City

**DOI:** 10.3390/ijerph16132318

**Published:** 2019-06-30

**Authors:** Ge Shi, Jie Shan, Liang Ding, Peng Ye, Yang Li, Nan Jiang

**Affiliations:** 1 School of Geographic Science, Nanjing Normal University, Nanjing 210046, China; 2 Lyles School of Civil Engineering, Purdue University, 550 Stadium Mall, West Lafayette, IN 47907, USA; 3 Key Laboratory of Virtual Geographic Environment, Ministry of Education, Nanjing Normal University, Nanjing 210046, China; 4 Jiangsu Center for Collaborative Innovation in Geographical Information Resource Development and Application, Nanjing Normal University, Nanjing 210023, China; 5 College of Computer and Information, Hohai University, Nanjing 210098, China; 6 Information Center of Jiangsu Natural Resources Department, Nanjing 210017, China

**Keywords:** urbanization, road network, PM2.5, spatial patterns, geographically weighted regression, Nanjing

## Abstract

Developing countries such as China are undergoing rapid urban expansion and land use change. Urban expansion regulation has been a significant research topic recently, especially in Eastern China, with a high urbanization level. Among others, roads are an important spatial determinant of urban expansion and have significant influences on human activities, the environment, and socioeconomic development. Understanding the urban road network expansion pattern and its corresponding social and environmental effects is a reasonable way to optimize comprehensive urban planning and keep the city sustainable. This paper analyzes the spatiotemporal dynamics of urban road growth and uses spatial statistic models to describe its spatial patterns in rapid developing cities through a case study of Nanjing, China. A kernel density estimation model is used to describe the spatiotemporal distribution patterns of the road network. A geographically weighted regression (GWR) is applied to generate the social and environmental variance influenced by the urban road network expansion. The results reveal that the distribution of the road network shows a morphological character of two horizontal and one vertical concentration lines. From 2012 to 2016, the density of the urban road network increased significantly and developed some obvious focus centers. The development of the urban road network had a strong correlation with socioeconomic and environmental factors, which however, influenced it at different degrees in different districts. This study enhances the understanding of the effects of socio-economic and environmental factors on urban road network expansion, a significant indicator of urban expansion, in different circumstances. The study will provide useful understanding and knowledge to planning departments and other decision makers to maintain sustainable development.

## 1. Introduction

Rapid urban expansion is an important period for developing countries due to the burgeoning economy as well as the road network expansion [1]. The world’s urbanization rate has increased from 39% to 52% for the last three decades and is expected to reach around 66% by the year 2050. The increase in urbanization in developing countries is much more rapid than in developed countries [2,3]. Managing growing cities is both critical and complex. Urbanization development greatly increases convenience for the residents but at the same time carries the risks of arable land shortage, over-population, traffic congestion, food security problems, environmental pollution, public health threats, and so on [4,5]. For this reason, research about the regulation of urban expansion has become even more significant in order to keep the city sustainable. The road network, the skeleton of the urban space structure, is an important driving force for space expansion and one of the key indicators of urban development [6,7]. Increasingly well-developed road networks have become common in modern cities recently [8]. A comprehensive road network is built to bring different regions closer and accelerate the information exchange between each space. However, the increasing road network cutting through the natural environment may bring about biodiversity loss, a series of environmental issues, and further facilitate urban sprawl [9]. Thus, road network development pushes local urban expansion to a certain degree; but on the other hand, it may also be a consequence of urban development [10,11]. It cannot be ignored that there is already a series of “city illnesses” due to rapid expansion, which have already had side effects on public health. In order to explore the mechanisms of urban expansion to deal with sustainable development, studying the patterns of road network expansion is a starting point. Remote sensing technology and a series of models enable researchers to map the urbanization process and the related social and environmental variables over vast areas.

Urban expansion regulation has been an important research topic for years, and scholars have studied it from multiple perspectives. Existing studies about urban expansion are mainly about economic development, built-up area growth, land use and cover change, and population growth, which have been investigated using government statistic year books, Landsat remote sensing images, and so on [12,13,14,15]. In more detail, the current studies are mainly of four types: (i) using remote sensing images to extract the spatial boundary of urban expansion [16,17,18,19]; (ii) using dynamic models to understand the spatiotemporal sprawl patterns [4,20,21]; (iii) using statistical models to uncover the mechanisms, effects, and driving forces behind urban expansion [22,23]; (iv) using prediction models such as the cellular automata model, agent based model, and clue model to simulate future development [24,25,26,27]. Moreover, researchers have usually focused on the regulation on a large-scale region from a macro perspective, and detailed studies about cities’ structural elements, such as road network spatial characteristics, and changing patterns are relatively rare.

As an important part of urban expansion, road network expansion has caught the interest of more scholars recently, as well as the secondary effects on ecology, urban landscape, land use and so on [28,29]. The road network of China has experienced rapid growth period over the last few decades, but theoretical research on it has only just been initiated in China, while it is widely studied in many other countries. As early as the 1960s, Garrison [30] used graph theory to describe the static spatial patterns of the transportation network from the perspective of geography, which method is still used in many studies nowadays. Yang [6] predicted the traffic flow amount to model the road network changes with a purpose to solve the mixed network design problem. Parthasarathi [31] and Levinson [32] used statistical methods to examine road network expansion based on the current traffic demand and road network data. Strano [33] characterized the road network growth pattern in the Groane area from 1833 to 2017 and found that the road network was expanding based on a pre-existing urban structure with a high centrality. Michael Betty’s group studied London’s road network development over a century from 1880 to 2013 by multiple methods to discover the growth patterns as well as the urban sprawl [34,35,36]. For example, a complexity network model was applied to characterize the topological and metrical patterns in a small scale region; space syntax was applied to discover the inner spatial interaction patterns of road network like centrality, movement accessibility, integration, etc. [37] and to explore the statistical laws between road network and urban sprawl. Rui and Ban [38] studied the relationship between road network density and land use type and found that they were highly correlated. Overall, studies on road networks have a long history with different perspectives, and the research results are temporal and regional. Yet, few studies have investigated regional road network evolution and its related social-economic and environmental influences locally. Chu used GWR to model the relationship between population and socio-economic growth described by nighttime light data [39]. Most existing researches about urban growth in China have focused on developed coastal cities and major metropolitan cities such as Beijing, Shanghai, Guangzhou, Shenzhen, and so on [40,41,42], and less attention has been paid to inland cities. However, urban expansion is a non-regular process over space and time, which means the urban expansion patterns may vary between different places and time periods [43]. Nanjing, one of the major cities of the Yangtze River Delta, is the capital city of Jiangsu province, and Jiangsu province is one of the largest economic development provinces of China. It has experienced a rapid development period recently, and its urban patterns change a lot. Some researchers studied Nanjing’s expansion regulation from different perspectives like land use changes and urban functional region changes [44,45,46]. However, the road network expansion and its variables regulation still need further investigation.

The primary objective of this research is to fill the gap in the previous research by exploring the regulation of urban growth during a rapid economic development period from the perspective of road network expansion and its potential social and environmental influences in Nanjing from 2012 to 2016. In doing so, the line density estimation model and other spatial analysis supported by ArcGIS (Esri Inc., Redlands, CA, USA) technology were used to extract the spatiotemporal patterns of the road network evolution. Then, the geographically weighted regression model was applied to discover the relationship between urban road network expansion and other social and environmental changes. The results and conclusions about the road network growth patterns and their potential influences aim to provide useful suggestions for planning authorities that need to make right decisions in order to maintain sustainable development during a fast urbanization and industrialization period in the same or similar regions with a rapid growth trend.

## 2. Material and Methods

### 2.1. Study Area

**Figure 1:**
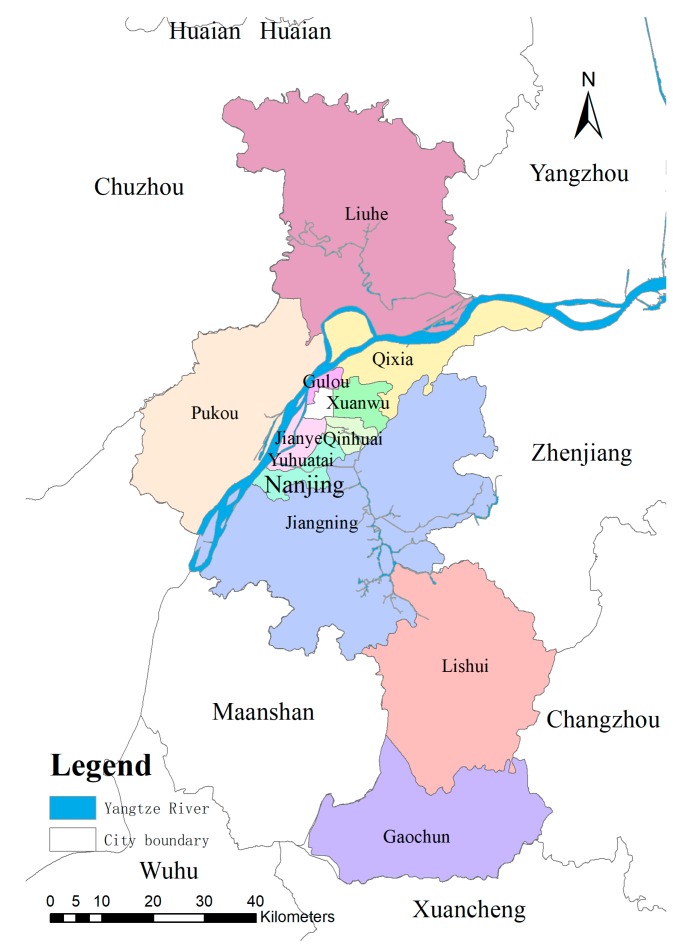
The research area and the distributions of administrative districts in Nanjing.

Nanjing, “Ning” for short, a city with more than 2000 years of history and an ancient capital city of six dynasties, is now the capital city of the Jiangsu province. Nanjing is an important political, economic, and cultural center and is the second largest city in the Yangtze River Delta region with a total area of 6597 km^2^. Nanjing is located in the south part of Jiangsu province in Eastern China in the northern subtropical climate zone with four well classified seasons and an annual average temperature of 15 °C. It is located between longitude 118°22′ E to 119°14′ E and latitude 30°14′ N to 32°37′ N (Figure 1). There are 11 administrative districts in the Nanjing metropolitan area divided into Jiangnan south of and Jiangbei north of the Yangtze River, running from southwest to northeast. Jiangnan district includes Xuanwu District, Qinhuai District, Jianye District, Gulou District, Qixia District, Yuhuatai District, and Jiangning District, while Jiangbei District includes Pukou District and Liuhe District. The remaining two districts located in the suburban area are Lishui District and Gaochun District. As of 2016, the permanent population is 8,335,000 of which the urban population is 6,858,900 or 82.3% of the total population. The regional GDP is 1282.04 billion Chinese Yuan [47]. There is an increasingly serious conflict between human social development and natural sustainability at this high urbanization level and rapid development speed.

### 2.2. Data and Preprocessing

• Road Network Data

This paper uses the road network data of Nanjing for 2012 and 2016, provided by the National Earth System Science Data Sharing Infrastructure (http://www.geodata.cn), Yangtze River Delta Science Data Center (http://nnu.geodata.cn:8008) [48], which serves as Nanjing’s basic geographic map. However, the data does not provide the road grades and types like artery roads or branch roads, thus the road network in this paper refers to all types of roads in Nanjing aggregated together. All images provided are under the WGS1984 coordinate system.

• City Boundary Data

This paper uses city boundary data of Jiangsu province from the National Science & Technology Infrastructure of China, National Earth System Science Data Sharing Infrastructure (http://www.geodata.cn) [48].

• Social and Economic Data

This paper uses Jiangsu Statistical Yearbook data and China City Statistical Yearbook data from 1996 to 2016, from the National Bureau of Statistics of China. This dataset is provided by the National Earth System Science Data Sharing Infrastructure (http://www.geodata.cn), Yangtze River Delta Science Data Center (http://nnu.geodata.cn:8008), which provides the Jiangsu province’s annual socioeconomic dataset [48]. This dataset includes statistical information of all cities in Jiangsu province and includes economic, social, demographic, agricultural, urban construction, environmental, and other related city statistics. This data reflects the yearly economic and social development statistics of Jiangsu province. The storage format is the Excel file.

• Particulate Matter 2.5 Concentration Data

There are 9 monitoring stations in Nanjing city, including the Olympic center station, Caochang gate station, Maigao bridge station, Pukou district station, Ruijin road station, Shanxi road station, Xianlin district station, Xuanwu Lake station, and Zhonghua gate station. The daily levels of PM2.5 at each station were obtained from the Ministry of Ecology and Environment (MEE) [49]. The annual averages of PM2.5 levels of each district in Nanjing were generated in ArcGIS (Esri Inc., Redlands, CA, USA).

### 2.3. Methods

#### 2.3.1. Line Density Estimation Model

To study the spatial distribution of the road network, we explored whether the road network distributed even across the city and where are the concentration points. To answer the above questions, the distribution map is hard to draw, thus, we employed a line density model to describe its concentration at a regional scale. The line density estimation model is used to describe density of the linear features in the neighborhood of the designed output raster cell. The length of the portion that falls within the raster cell as the total road network considered in this unit was calculated [50]. The calculation formula is as follows:

(1) $$LD_{i}=\frac{\sum_{1}^{n}L_{k}}{A}$$

where, $LD_{i}$ is the road network line density in year i; L stands for the length of the portion of roads that falls in the unit k; and A is the area of total unit.

#### 2.3.2. Road Network Expansion Rate

The road network expansion rate of Nanjing city was measured by calculating the total change of road network in the study area. This index shows the average annual growth rate of the urban road network over the study period. If this value is above zero, the corresponding research period is experiencing an expansion time. Otherwise, if the value is below zero, it is experiencing a loss period. The calculation formula is shown as below:

(2) $$ER_{}=\frac{RL_{n+i}-RL_{n}}{i}$$

where ER refers to the road network expansion rate from year n to year n+i; $RL_{n+i}$ refers to the total length of road network in year n+i in kilometer; and ${\mathrm{RL}}_{n}$ refers to the total length of road network in year n in kilometer.

#### 2.3.3. Geographically Weighted Regression (GWR) Model

The previous studies discovered that urban expansion and environmental issues have a significant correlation but vary over different regions and changes with spatial context to various degree. The geographically weighted regression (GWR) model is widely used to explore the spatial variables [51]. Compared with other traditional widely used linear or nonlinear regression models, the GWR model adds the spatial variation in this regression model coefficients, which resulted in improving the estimation accuracy significantly. As we discovered in this study, previous studies have shown a lot about the correlations between road network expansion and human–land related elements, they differ in different districts. Therefore, it is necessary to estimate the local level concentration of road network growth based on social and environment variables. This study considered the GDP of first second and third industries, PM2.5 concentration level, and population as the most affected elements of urban road network expansion, as the parameters entered into the GWR model. The GWR model estimates the parameter for a given location ($u_{i},v_{i}$) by a weighted least squares method, expressed as follows:

(3) $$y_{i}=\delta_{0}\left(u_{i},v_{i}\right)+\overset{p}{\underset{k=1}{\sum }}\delta_{k}\left(u_{i},v_{i}\right)x_{ik}+\mu_{i}$$

where, $y_{i}$ is the dependent variable change of road network; $x_{ik}$ (k = 1, 2,…, 5) are the independent variables, including GDP of agriculture, GDP of industry, GDP of service sector, PM2.5 concentration level and population; $\left(u_{i},v_{i}\right)$ is the spatial coordinate of sample point i; $\delta_{k}\left(u_{i},v_{i}\right)$ stands for the regression parameters of sample point i (i = 1, 2, 3,……, n); and $\mu_{i}$ is the random error of each individual sample point. In the process of modeling, the bandwidth is an important parameter to control the degree of smoothing; we applied the adaptive kernel method to decide the bandwidth [52,53].

## 3. Results

### 3.1. Spatial Patterns of the Road Network

**Figure 2:**
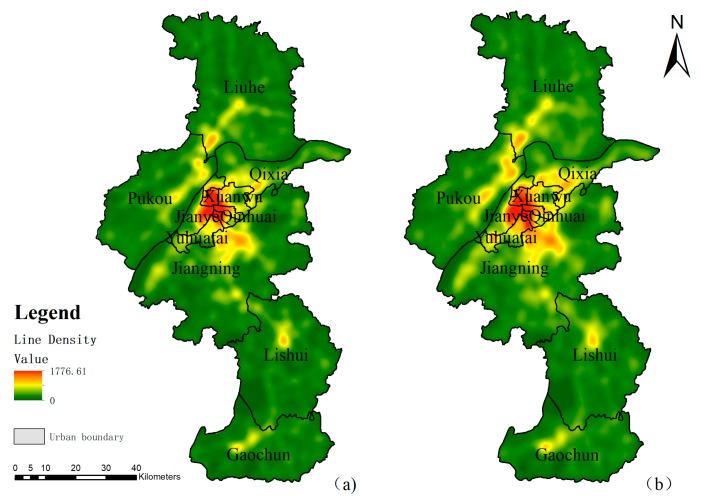
Line density of the road network in Nanjing. (**a**) Line density of road network in 2012; (**b**) Line density of road network in 2016.

The road network distribution of Nanjing in 2012 and 2016 was taken as the case study. The spatial distribution patterns of the road network are generated based on the line density estimation model (Figure 2). It revealed that the distribution is uneven across the city, and there is obvious concentration to the south of the Yangtze River, with some of the densest regions spread along the Yangtze River, and then gradually dispersed to the periphery. The overall distribution of the road network shows a morphological character of two horizontal and one vertical concentration lines. There is no doubt that the downtown area has the highest road network density, which was mainly distributed in Xuanwu district, Qinhuai district and the east part of Jianye district in the year 2012. It is clear to see the high density district expanded until 2016, mainly around the former high-density region. In the suburban regions like the Liuhe district, Lishui district, and Gaochun district there are some small-scale aggregations. The expansion trend is obvious by comparing the two period’s maps, and a deeper comparison of the changes will be analyzed in the next section.

### 3.2. Expansion Patterns of the Road Network

**Figure 3:**
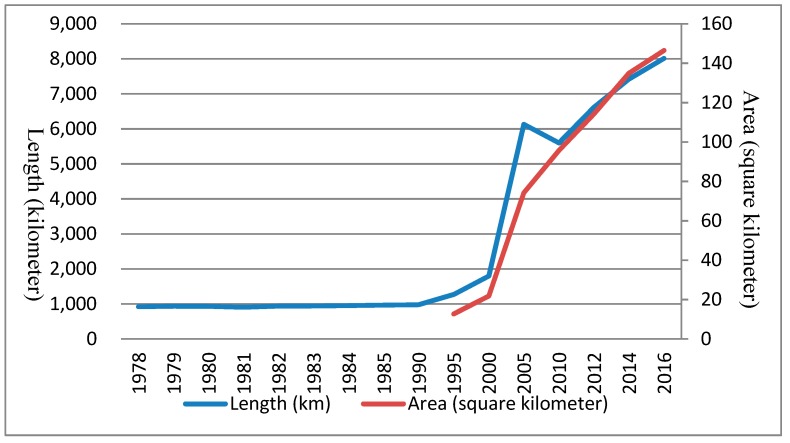
The length and area of road network in Nanjing.

Using Nanjing Yearbooks since 1978 [54], we found information about the construction of the road network (Figure 3). China has experienced a rapid social and economic development since the Reform and Open Program was established in 1978. Urban infrastructure construction has also undergone a correspondingly large development in order to meet the increasing social need. Through this time period, the construction of the road network in Nanjing increased, while in 1980 and 1981 it slightly recessed because the agriculture industry was the support industry at that time, and arable land accounted for the major land use type. From 1982 to 1990, the road network construction began to slowly increase, while economic development also occurred at a low pace. Then, urbanization construction began to take place and rapidly expanded until 2005, with an average road length growth rate of 866 km per year and an area expansion rate of 10,484,000 m^2^ per year. From 2005 to 2010, the length of the road network decreased at a rate of 106.6 km per year, as the urbanization rate tended to reduce during that period. During the same time period, the city-loop expressway underwent upgrade construction, resulting in the reduction of its length, and some transportation land was converted into other types of land use under the Intensive Land Use policy [20,55]. However, Nanjing City was still undergoing a huge urbanization and infrastructure construction period, thus, the area of the road network still expanded at a speed of 4.2908 million m^2^ per year. Since 2010, urban construction has been characterized as being in a maturity phase [56], the expansion rate of the road network has reduced but still increases by hundreds of kilometers per year as is the area of the road network. From 2012 to 2014, the length growth rate decreased but the area growth rate increased as construction mainly focused on widening the existing roads, rather than adding new roads. From 2014 to 2016, both the length growth rate and the area growth rate decreased by more than 25% as urban construction became sophisticated and road network construction gradually became stable. The spatial patterns of road network development from 2012 to 2016, this relatively developed period, will be focused on in the following sections.

**Figure 4:**
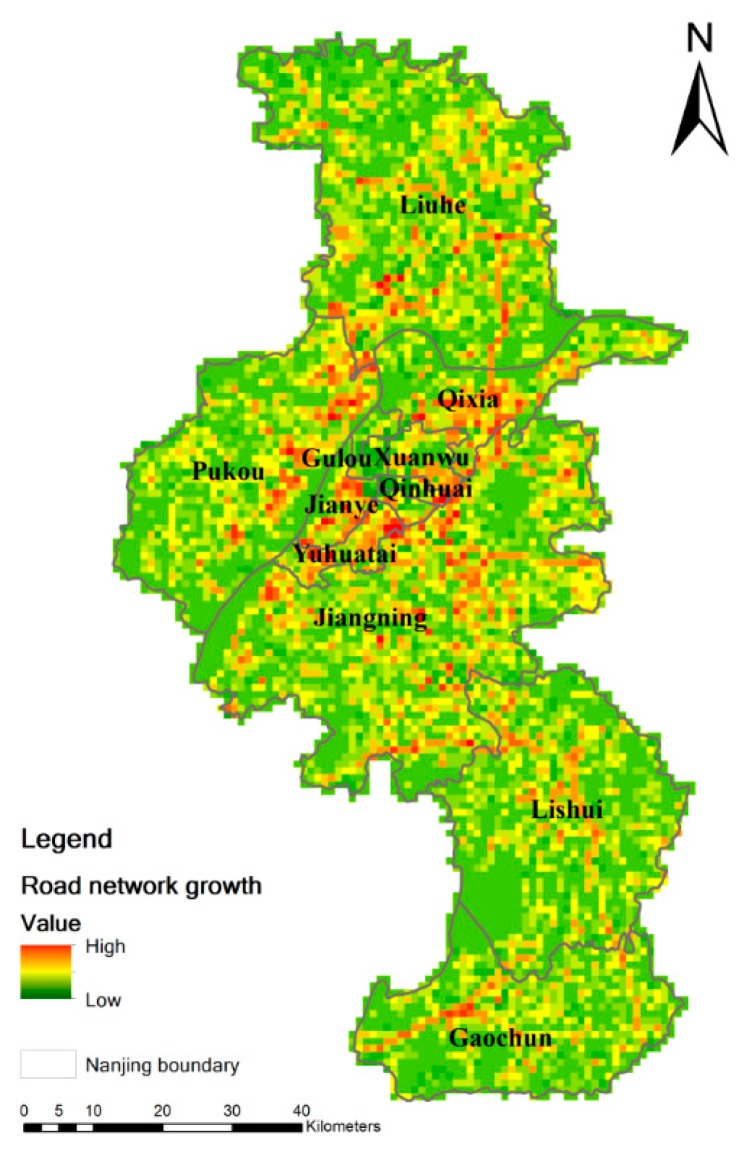
Spatial distribution of road network growth in Nanjing from 2012 to 2016 (Unit: km/km^2^).

From 2012 to 2016, the road network expanded by 1397.07 km in length and 32.2477 million m^2^ in area. To further analyze the two phases of road network maps, we employed the spatial analysis of ArcGIS software to find out the spatial distribution of expansion of the road network from 2012 to 2016 (Figure 4). The spatial distribution map reveals that there was uneven expansion across the city to different degrees. To be specific, the metropolitan center (Gulou District, Xuanwu District, and Qinhuai District) had relatively low expansion, while the sub-centers (Pukou District, Qixia District, Lishui District, and Gaochun District) and the surrounding downtown area (Jianye District, Yuhuatai District, and the southern part of Qinhuai District) had significant expansion concentration. The concentration along the major high way was also obvious in order to make the connection with local regions closer.

### 3.3. Socio-Economic and Environmental Development of Nanjing

Rapid urban expansion in a short period may bring about numerous “urban illnesses”, such as shortage of natural resources, environmental pollution, urban transportation congestion, raising living cost, and so on [57]. Thus, understanding the urban system is becoming important. In general, road network expansion is probably caused by social factors or government department decisions, affecting social development. Usually, the factors not only affect road network expansion but are also affected by the road network system, as well as environmental issues. Road network development and social and environmental factors influence each other to a different degree. In this study, we consider the social factors of economic development and population and the environmental factor of air pollution as the most important factors when analyzing the influencing factors within a short time scale, which will be discussed by spatial analysis in more detail below.

**Figure 5:**
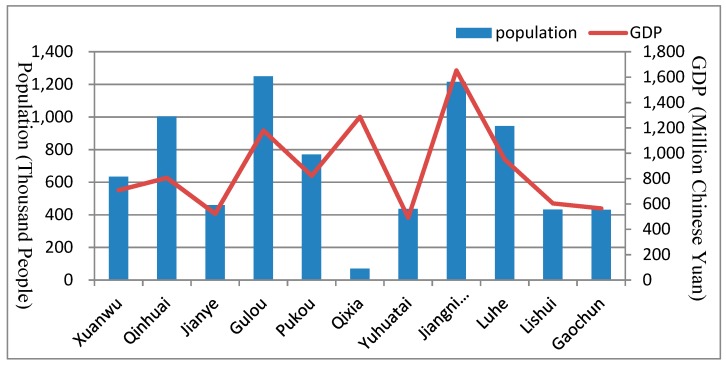
Population and total GDP.

**Figure 6:**
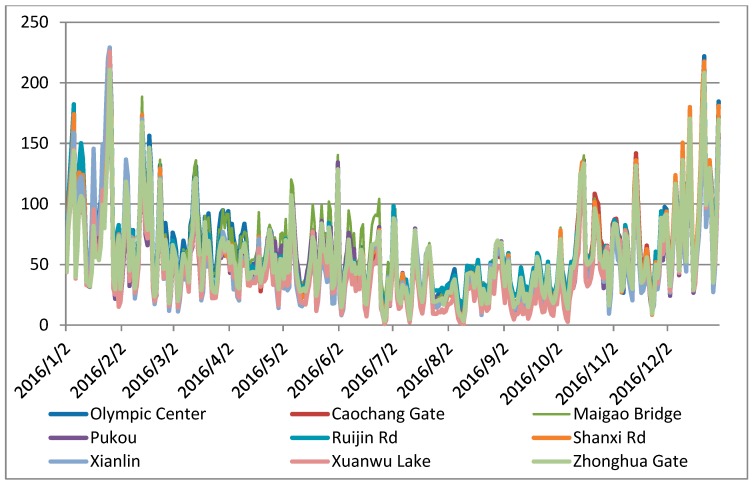
Value of PM2.5 of each site in 2016 unit: μg/m³.

According to the census report and the Nanjing Yearbook in 2016 [54], the GDP information and population of each district in Nanjing are shown in Figure 5. In the downtown region, GDP has a high linear correlation with the population. However, in the suburban area of Qixia District and Jiangnin District, a high GDP with a low population is seen, which is caused by the distribution of heavy industry. Based on the environmental report [49], the major air pollution in Nanjing is the PM2.5 concentration. Thus, we collected the PM2.5 concentration data from 2016 to describe the air quality of each district in Nanjing. There are nine air quality sensor stations in Nanjing, which are the Olympic Center station, Caochang Gate station, Maigao Bridge station, Pukou station, Ruijin Road station, Shanxi Road station, Xianlin District station, Xuanwu Lake station, and Zhonghua Gate station. Although air pollution should be determined by spatially continuous information on PM2.5 concentration, in this study we used the sensor station data to describe the air quality situation in the observed districts. The PM2.5 concentration of each districts was calculated (Figure 6), and we found that there is a strong correlation between different districts, and the records in the downtown area are higher than those in suburban regions. This high PM2.5 level can not only be the result of population concentration, traffic demand growth, new construction, human activity concentration, and so on but also are the reason of the above elements. Thus, the PM2.5 level is both the reason and the cause of the road network expansion.

**Figure 7:**
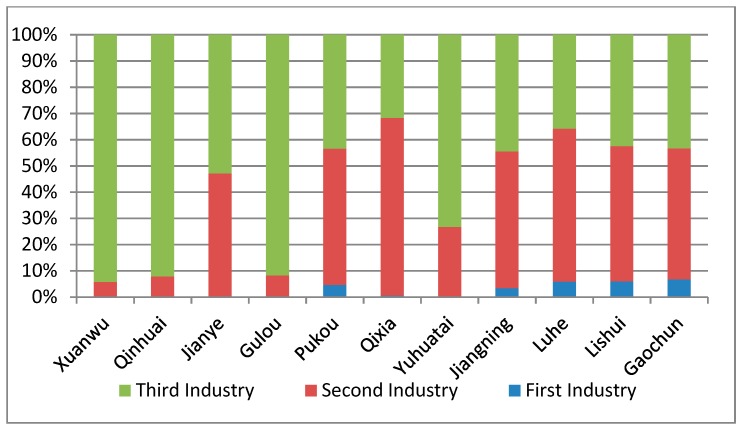
The industrial structure of each district in Nanjing.

**Figure 8:**
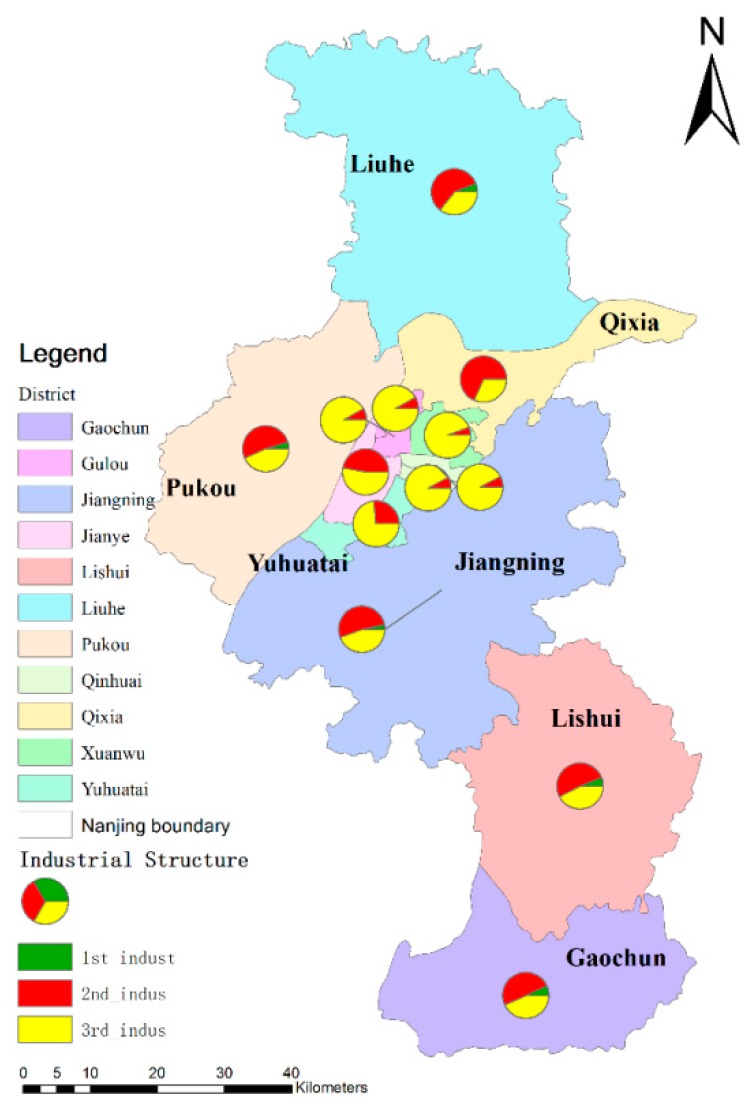
The spatial distribution of industrial structure of each district in Nanjing.

Industry structure (see Figure 7 and Figure 8) is another important symbol to express the local development situation. Nanjing has experienced rapid economic development, and the industrial structure is also constantly being optimized. From 1978 to 2016, the proportion of first industry (agriculture) decreased rapidly, and some districts like Xuanwu District, Qinhuai District, Jianye District, Gulou District Yuhuatai District, and Qixia District in the metropolitan area have no first industry. The proportion of the second industry (industry) also decreased a bit, while the proportion of the third industry (services) continued to improve, especially in the downtown region. Until 2016, the industrial structure of Nanjing city had formed a situation in which services were the main contributors in metropolitan districts; industries and services were the main contributors in suburban districts; and agriculture was a supplementary industry.

### 3.4. Road Network Expansion in Relation to Its Driven Variables

**Figure 9:**
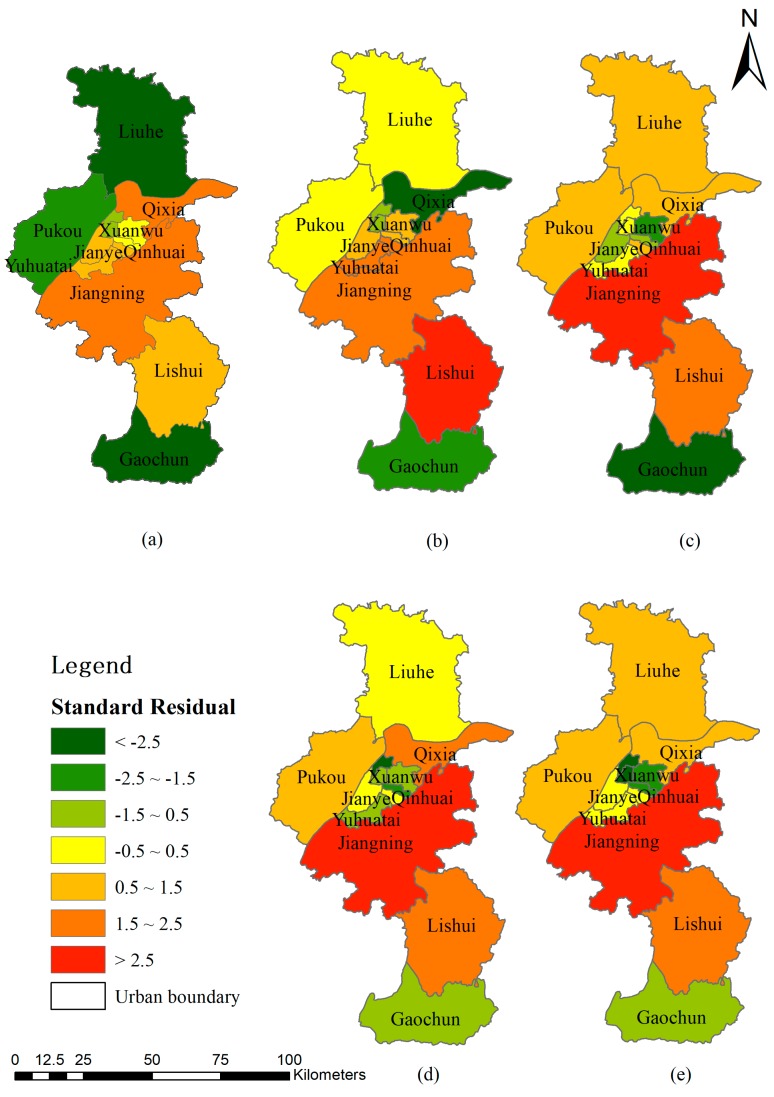
The standard residual map of the relationship between road network expansion and selected variables by geographically weighted regression. (**a**) The first industry; (**b**) The second industry; (**c**) The third industry; (**d**) PM2.5 index; (**e**) Population.

With the support of ArcGIS technology, we built a regression model between road network expansions from 2012 to 2016 and other variables separately to determine whether there are some districts that have a weak correlation with the variables. According to the model result, the local R^2^ and adjusted local R^2^ are all higher than 0.7. The value of the standard residual for road network expansion and each variance were mapped (see Figure 9). We observed that some districts have very high residual values (higher than 2.5) with unusual explanations. To be specific, Lishui (see Figure 9b), located in the southern part of Nanjing, is an important transportation hub and logistics center in the East China region. The major economic support there is based on agriculture, tourism and new-type industrial parks. From the above analysis, road network expansion in Lishui was found to lag behind the other districts a lot as this region already has a sufficient transportation network to support the local industry. Therefore, the distribution of road network expansion cannot be explained well by the development of the second industry in Lishui. Jiangning District, located to the south of the downtown area, was also found to have a high standard residual value of the third industry, PM2.5 concentration and population density. Due to the Nanjing Comprehensive Planning 2020, Jiangning District is one of the major new-built residential areas, which was planned in order to release the pressure from the downtown area, where the population density is relatively high [56]. Thus, the expansion of the road network in Jiangning District may mainly be a result of policy changes. Moreover, there is more green land and natural resource in Jiangning, and thus the air quality is better than in the downtown area. The development of the third industry is stable without significant change. Thus, the distribution of road network expansion in Jiangning District cannot be well explained by the third industry, PM2.5 concentration or population density. Overall, the districts with normal standard residual values that range from −2.5 to 2.5 are the majority of the whole Nanjing city, which reflects that the correlations between road network expansion and air quality, industrial structure, and population density are stable.

To further investigate the relation between road network expansion and the variables, the multi-variance GWR was computed. The value of local R^2^ and the adjusted local R^2^ were 0.812 and 0.815, respectively, which are larger than each of the individual GWR models above. The local models showed statistical significance with the p value lower than 0.05 Plus, the standard residuals were all below 2.5, which shows that this model performs well with the supposed variables.

The coefficient and standard error (see Table 1) show a more detailed relationship. The computed results demonstrate that the first industry and second industry have a positive correlation with the road network expansion, while the third industry, PM2.5 concentration, and population density have a negative correlation. The second industry has the largest coefficient among all the variables. Therefore, the second industry accounts for the major cause of road network expansion; this is because, as the local governments develop the economy, more convenient transportation is needed to further promote it. The first industry accounts for the second cause, because the region with more agricultural area is usually considered as the less developed region with a low road network density. There is no arable land in downtown Nanjing with a higher road network expansion rate. Therefore, the less organized region with more arable land may have more road network construction. The same situation applies with the population density variable. Although the district with a higher population may have a demand for a larger road network, there is a certain time lag between the interaction, and the policy plan has a large impact on the project construction. Nanjing city has experienced rapid economic and urban growth and is now the core region of the Yangtze River Delta region. Its road network expansion pattern and influences should be carefully considered to maintain sustainable development.

## 4. Discussion

Most cities have experienced a rapid urbanization period as well as economic development since the Reform and Opening up policy in 1978, especially the developed cities in Eastern China like Nanjing. This rapid urbanization has been greatly attributed to the road network expansion. To reduce over-construction and plan properly, we investigated the spatial patterns of the road network and its expansion trend and applied the GWR model to find out the correlation between the social and environmental influences. Most of the associations between the road network and other social and environmental influences considered are as expected. Therefore, there is sufficient reason to consider that during the recent developing period in Nanjing, the places with higher first and second industries and relatively lower third industry percentage and population and less air pollution would have more road network expansion.

In China, road network construction is primarily based on planning by the transportation department, mainly according to the research about regional accessibility [58,59]. Human activity, environmental changes and economic development all have a strong impact on road network expansion but at different scales with the different regions’ local development plans. Therefore, some districts with low population may have high rates of road network expansion, while the high-density districts with a large GDP value could have lower expansion rates for multiple reasons. In Nanjing, where the metropolitan areas have a large population, high GDP value, relatively higher PM2.5 concentration, and large proportions of third industry, the road network is already extensive, thus, it has had a relatively low expansion rate in the recent study period. However, looking at the suburban centers in Nanjing (Jiangning District, Lishui District, Gaochun District, Qixia District, Liuhe District and Pukou District), the proportion of first and second industries are both high, and population density is relatively low, and their road network expansion rate showed an increasing trend recently. In addition, the air quality, GDP values, industry structure, and population growth are not only the influencing factors of road network expansion but are also the result of it. They influence each other at different scales in different aspects [60]. From the perspective of urban management, the road network is a complex system, which impacts humanity’s daily lives directly. The existing research mainly explores the quantity spatial patterns, whereas this study provides a method of using the geographically weighted regression model for looking into the influencing factors. This correlation provides effective measures for policy makers to consider the balance between new urban construction and existing development to reduce the waste and potential negative effects.

## 5. Conclusions

In this study, taking the road network of Nanjing for 2012 and 2016 as an example, we analyzed the temporal and spatial patterns of the road network distribution, its dynamic patterns, and potential social and environmental influences through a mathematical and spatial model. To be specific, we analyzed the road network line density spatially, road network expansion rate, and the correlation between road network expansion and the industry structure, PM2.5 concentration and population distribution. From the discussions above, the following main conclusions were reached:

(1) The distribution of the road network appears uneven in Nanjing, where the downtown area has the highest road network density, which decreases out to the periphery. The Yangtze River divides the city and the road network concentration into two clear parts. In the southern part, there are one clear downtown center and two sub-centers. The overall layout shows a morphological character of two horizontal and one vertical spatial road network distributions.
(2) Since 1990, the expansion of the road network has occurred to different degrees across Nanjing City. The expansion continues while the expansion rate has declined since 2012. From 2012 to 2016, the expansion mainly happened in the suburban area near the downtown region due to the new district construction planning.
(3) The GWR spatial analysis model enables us to discover that, apart from the policy issue, the first industry and the second industry significantly promote road network expansion, while the third industry, PM2.5 concentration, and population have a negative correlation. Among the aforementioned industries, the second industry was the most significant influencing factor for the road expansion during the research period. The road network expansion is mainly affected by the second and third industries, PM2.5 concentration, and population density in the metropolitan region and by first and second industries in the suburban region. This is not only the result of urbanization but also reflects the planning decision making.

This paper presents a comprehensive insight into the spatiotemporal patterns of road network distribution and expansion in Nanjing City at a district scale for the first time. Nanjing, one of the core cities in the Yangtze River Delta city circle in the eastern coastal region of China, has a rapid development speed and will continue to maintain a superior social and economic development trend in the near future. The road network is a complex system and performs as the skeleton of the city, which needs to be carefully studied to maintain sustainable development. This finding has implications for future transportation planning based on the road network patterns discussed above. In a future study, we should focus on promoting the usage efficiency of the existing road system and seek development under the framework of social and environmental benefits.

## Figures and Tables

**Table 1:** Key parameters derived from the GWR models with combination explanatory variable.

Variables	Coefficient	Standard Error (Unit: km)
First industry	0.32	0.024
Second industry	0.47	0.001
Third industry	−0.15	0.001
PM2.5 concentration	−0.29	0.021
Population density	−0.17	0.028
